# The effectiveness of non-surgical intervention (Foot Orthoses) for paediatric flexible pes planus: A systematic review: Update

**DOI:** 10.1371/journal.pone.0193060

**Published:** 2018-02-16

**Authors:** Sindhrani Dars, Hayley Uden, Helen A. Banwell, Saravana Kumar

**Affiliations:** School of Health Sciences, Sansom Institute for Health Research, University of South Australia, Adelaide, Australia; Bern University of Applied Science, SWITZERLAND

## Abstract

**Background:**

Flexible pes planus (flat feet) in children is a common presenting condition in clinical practice due to concerns amongst parents and caregivers. While Foot Orthoses (FOs) are a popular intervention, their effectiveness remains unclear. Thus, the aim of this systematic review was to update the current evidence base for the effectiveness of FOs for paediatric flexible pes planus.

**Methods:**

A systematic search of electronic databases (Cochrane, Medline, AMED, EMBASE, CINHAL, SportDiscus, Scopus and PEDro) was conducted from January 2011 to July 2017. Studies of children (0–18 years) diagnosed with flexible pes planus and intervention to be any type of Foot Orthoses (FOs) were included. This review was conducted and reported in line with the Preferred Reporting Items for Systematic Reviews and Meta-Analyses (PRISMA) statement. McMaster critical review form for quantitative studies, was used to assess the methodological quality of the included studies. Given the heterogeneity of the included studies, a descriptive synthesis of the included studies was undertaken.

**Results:**

Out of 606 articles identified, 11 studies (three RCTs; two case-controls; five case-series and one single case study) met the inclusion criteria. A diverse range of pre-fabricated and customised FOs were utilised and effectiveness measured through a plethora of outcomes. Summarised findings from the heterogeneous evidence base indicated that FOs may have a positive impact across a range of outcomes including pain, foot posture, gait, function and structural and kinetic measures. Despite these consistent positive outcomes reported in several studies, the current evidence base lacks clarity and uniformity in terms of diagnostic criteria, interventions delivered and outcomes measured for paediatric flexible pes planus.

**Conclusion:**

There continues to remain uncertainty on the effectiveness of FOs for paediatric flexible pes planus. Despite a number of methodological limitations, FOs show potential as a treatment method for children with flexible pes planus.

**PROSPERO registration number:**

CRD42017057310.

## Introduction

Pes planus, commonly known as flat feet, describes feet with lowered medial longitudinal arches [1, 2]. Pes planus can be classified into two types, rigid and flexible, if the arch reforms in non-weight bearing compared to weight-bearing it is considered flexible pes planus which is often associated with hindfoot (also known as rearfoot) eversion [3, 4]. If the arch height does not change during non-weight bearing compared to weight-bearing it is classified as rigid pes planus which affects around 1% of the population [1, 5]. While paediatric flexible pes planus is common, affecting around 48% to 77.9% children [6–8] there is ambiguity in terms of its definition, diagnosis and management strategies. The prevalence estimates of flat feet within the literature, whilst inconsistent, suggests that it is more frequently seen in younger children, males and those who are overweight or obese [7, 9, 10].

Paediatric flexible pes planus is further categorised as symptomatic and non-symptomatic, with the later subdivided into developmental (arch develops with age) and non-developmental (arch does not develop with age) [1, 5]. From a clinical practice perspective, there is no single universally accepted diagnostic technique [11] to diagnose flexible pes planus. Instead the diagnosis relies upon a plethora of diagnostic techniques in both weight bearing and non-weight bearing positions inclusive of: rearfoot angle, heel position (valgus/varus), navicular height and arch formation [1, 3]. Individually, these assessments have been shown to have either low, moderate or untested diagnostic accuracy [12]. Recently, the paediatric flatfoot proforma (p-FFP) has attempted to standardise diagnoses, and direct when intervention is required, using a combination of subjective assessment points and a range of foot posture measures [5]. The uptake of use of the p-FFP by clinicians remains unknown and the proforma does not direct specific management techniques.

A range of management approaches have been proposed for paediatric flexible pes planus. Some studies have highlighted that treatment may be unnecessary, suggesting asymptomatic flatfeet do not decrease motor ability, sports performance or cause disability [3, 10, 13]. Conversely, other researchers suggest that paediatric flexible pes planus can cause pain, abnormal gait, poor balance, motor dysfunction and activity withdrawal thus justifying intervention [1, 7, 14]. Furthermore, as symptoms may continue into adulthood, podiatric management may be required [1, 15].

A range of non-surgical interventions have been proposed including activity modification, weight reduction, joint manipulations, serial casting, and stretching exercises [1, 6, 14]. The most frequently cited podiatric intervention for flexible pes planus, however, is foot orthoses (FOs) [2, 16–18]. Despite numerous research investigations, the evidence for the use of FOs in treating paediatric flexible pes planus remains equivocal [5, 14, 19, 20]. The most recent systematic review on this topic was undertaken by MacKenzie et al. (2012), who were unable draw any definitive conclusions on the efficacy of FOs due to the heterogeneity and methodological bias in the included studies [20]. Similarly, a Cochrane review in 2010 also resulted in a similar conclusion [14]. Given that the search for the previous systematic review was undertaken in 2011, it is timely to update the evidence base for this topic. Therefore, the aim of this systematic review was to identify the effectiveness of FOs for paediatric flexible pes planus.

## Methods

### Search protocol and registration

A protocol for this systematic review was registered at the international prospective register of systematic reviews–PROSPERO (Registration # CRD42017057310).

### Search strategy

This review was conducted and reported in line with the Preferred Reporting Items for Systematic Reviews and Meta-Analyses (PRISMA) statement [21] (S1 Appendix). Eight electronic databases were searched from January 2011 to July 2017 including: Cochrane Central Register of Controlled Trials (CENTRAL), Ovid Medline, Allied and complementary medicine–AMED, Ovid Embase, The Cumulative Index to Nursing and Allied Health Literature–CINAHL, SportDiscus, Scopus and Physiotherapy Evidence Database–PEDro. The following search terms were used with truncation and MESH headings where relevant: Pesplanus, Pes planus, Planovalgus, flat feet (flat f??t), low arch, ortho*, insoles, shoe inserts, treat*, non-surgical, therap*, interven*, manage* and effic* and rehab*, child*, toddler, kid*, teen*, juvenile, and adolescent. The search was limited to humans, English language, publication year from 2011 onwards, and age (0–18 years). An example search of Ovid AMED is outlined (S2 Appendix). Secondary search was performed on reference lists, cited by similar or recommended articles sections in different databases.

### Study designs

All forms of primary research designs were considered including randomised controlled trials (RCTs), clinical control trials (CCTs), quasi-experimental, pre-post cohort studies and case studies. The eligibility criteria for the population-intervention-comparator-outcome (PICO) is outlined below.

### Population

The studies were included if the participants were children (aged 0–18 years) of either gender diagnosed with flexible pes planus, irrespective of the diagnostic criteria used. Studies were excluded if participants had any history of injury or surgery of the lower limbs or conditions affecting lower limbs including infectious or systemic conditions, muscular, neurological or osseous abnormalities.

### Intervention

Studies were included if the intervention was any type of foot orthoses. This may include customised or pre-fabricated orthoses with any specific characteristics and modifications.

### Comparator

The acceptable comparators were control (no intervention provided) or alternate interventions (shoes, physical therapy, exercise, manipulation and/or acupuncture).

### Outcome

Due to a variety of outcomes related to the effects of FOs on pes planus, the search was not limited to any specific outcomes. Outcomes of interest included but were not limited to pain, function, self-perception, static foot posture and kinematics of gait.

### Literature search

Following development of the search strategy, a review protocol was established and databases searched. All search results were pooled and duplicates were removed. Titles and abstracts were screened before analysing the full texts to determine their eligibility. Two reviewers (SD and SK) independently assessed relevant studies to be included based on the eligibility criteria. Any disagreements were resolved by discussion with a third reviewer (HB), where required.

### Methodological quality

The McMaster Critical Review Form for Quantitative Studies [22] was used following the guidelines [23] to assess the methodological quality of the included studies. This tool assessed eight main components including: study purpose; literature review; study design (all experimental designs); sample (participants’ description, size justification, ethics and consent); outcomes (reliability and validity, outcome areas and measures used); intervention (description, contamination and co-intervention); results (statistical and clinical significance, analysis methods and drop outs) and conclusion with implications to practice (limitations and biases). To suit this review, the McMaster Critical Review Form for Quantitative Studies was modified to include questions on the randomisation of groups where relevant, and the reliability of the assessment methods used to establish the diagnosis of paediatric flexible pes planus. The individual components were rated as ‘yes’, ‘no’, ‘not-addressed’ or ‘NA–Not Applicable’. A score of ‘1’ was given to ‘yes’ and ‘0’ to ‘no and not-addressed’ while if ‘NA’ category applied then the total scoring was changed accordingly. The total score depended on the research design and relevant components with the maximum score being 17 (S3 Appendix).

Three reviewers (SD, HB and SK) independently assessed the methodological quality of the included studies and any disputes were resolved through discussion. To determine the level of evidence of included studies, the Intervention category of the Australian National Health and Medical Research Council’s (NHMRC) evidence hierarchy was used [24].

### Data management

Data were extracted by three independent reviewers using Microsoft Excel Spreadsheets (Microsoft Corp, Redmond Washington, USA) customised for this systematic review, any disputes were resolved through discussion. Data extracted included study and participants’ characteristics, interventions, comparators and outcomes. Additional data extracted included study’s protocol, diagnostic measures used for pes planus, measures of outcomes and adverse outcomes (S3 Appendix). Studies were categorised based on the types of orthoses used, outcomes measured and results compared. Due to the heterogeneity of the included studies, a meta-analysis was not conducted. Instead a descriptive synthesis of the results was undertaken.

### Synthesis of results

The NHMRC FORM methodology [25] was used in the interpretation of findings and the implications for clinical practice. Previous systematic reviews have used this framework successfully [26, 27]. The framework consists of five main components: 1) evidence base (level on evidence hierarchy); 2) consistency; 3) clinical impact; 4) generalizability; and 5) applicability to the Australian health care setting. The applicability component was not used for this systematic review due to its international focus.

## Results

### Study selection

**Fig 1:**
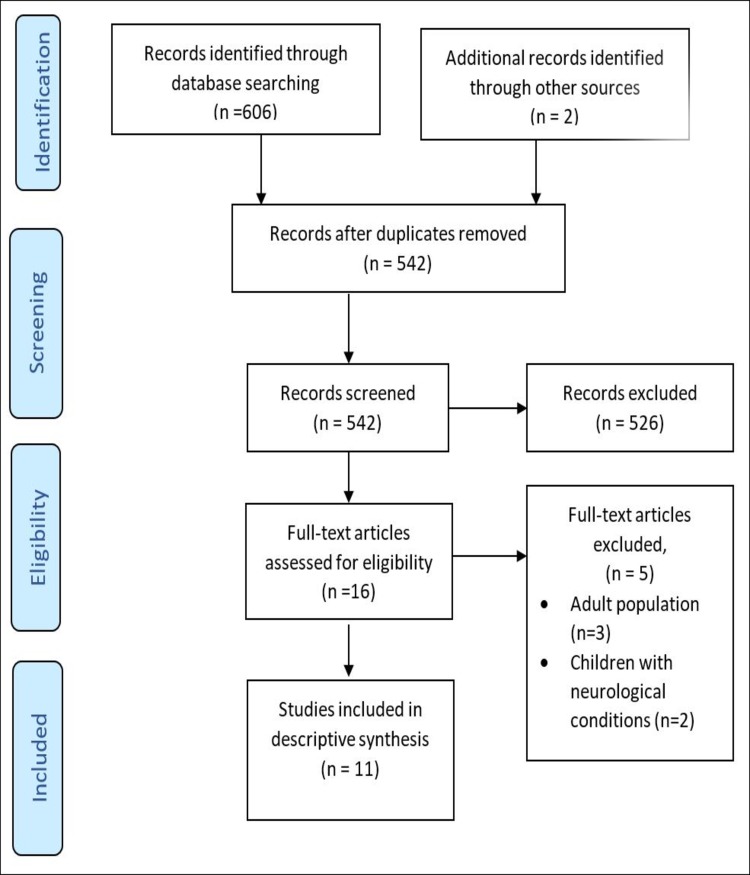
PRISMA flow chart of selection criteria.

The initial search identified 606 studies. After pooling the searches and removing the duplicates, 542 articles were screened for titles and abstracts. Sixteen studies were reviewed in full and 11 successfully met the eligibility criteria. Five studies were excluded as they recruited adult participants (n = 3) and children with neurological conditions (n = 2). The literature selection process is outlined in Fig 1.

### Risk of bias

Table 1 provides an overview of the levels of evidence and critical appraisal scores of the included studies. As per NHMRC levels of evidence, three studies were rated as Level II (RCTs) [28–30], two studies as level III-2 (Case-controls) [31, 32] and six studies were rated level IV (five case-series [33–37] and one single case study [38]). The main methodological concerns amongst the included studies were: lack of justification of the sample size (only one study did the power calculation [36]); lack of clear diagnostic criteria on how pes planus was diagnosed (only five studies cited their criteria for diagnosis [30, 32, 35–37]); lack of psychometrically robust outcome measures [OMs] (validity and reliability recorded in one study [35] and four studies reporting validity only [29, 31, 33, 38]); lack of detailed descriptions regarding intervention (only six studies provided adequate description of the FOs used [33–38]); and lack of reporting of clinical significance (only two studies included the effect sizes [36, 37]). Furthermore, the method of randomisation was not mentioned within the three RCTs and two studies failed to adequately detail the ethics approval and participants’ consent [9, 13].

**Table 1:** Levels of evidence and modified McMaster results of methodological quality.

Study	NHMRC level and study design	Items on modified McMaster critical review form																	Raw score and %
		1	2	3a	3b	3c	3d	3e	4a	4b	5a	5b	5c	6a	6b	6c	6d	7	
Asgaonkar and Kadam [28]	II; RCT	Y	Y	Y	N	Y	NA	NA	NA	NA	N	Y	Y	Y	Y	N	Y	Y	10/17 59%
Sinha et al. [29]	II; RCT	Y	Y	Y	N	Y	NA	N	NA	Y	N	N	Y	Y	Y	N	Y	Y	10/17 59%
Pandey et al. [30]	II; RCT	Y	Y	N	N	Y	N	Y	NA	NA	N	Y	Y	N	N	N	Y	N	7/17 41%
Pauk and Ezerskiy [31]	III-2; Case-control	Y	N	Y	N			NA	NA	Y	N	Y	Y	Y	Y	N	Y	N	8/15 53%
Aboutorabi et al. [32]	III-2; Case-control	Y	Y	Y	N			Y	NA	NA	N	Y	Y	Y	Y	N		Y	9/14 64%%
Bok et al. [33]	IV; Case series (pre+post)	Y	Y	Y	N			NA	NA	Y	Y	Y	Y	Y	Y	N	Y	Y	11/15 73%
Lee et al. [34]	IV; Case series (pre+post)	Y	Y	Y	N			NA	NA	NA	Y	Y	Y	Y	Y	N	Y	Y	10/15 67%
Bok et al. [35]	IV; Case series (post-test)	Y	Y	Y	N			Y	Y	Y	Y	Y	Y	Y	Y	N		Y	12/14 86%
Jafarnezhadgero et al.(a)[36]	IV; Case series (post-test)	Y	Y	Y	Y			Y	NA	NA	Y	Y	Y	Y	Y	Y		Y	12/14 86%
Jafarnezhadgero et al.(b) [37]	IV; Case Series (post-test)	Y	Y	Y	N			Y	NA	NA	Y	Y	Y	Y	Y	Y		Y	11/14 78%
Su et al. [38]	IV; Single Case study	Y	N	N	N			NA	NA	Y	Y	Y	Y	N	Y	N		Y	7/14 50%

McMaster items to be scored: 1. Was the purpose stated clearly?; 2. Was relevant background literature reviewed?; 3a. Was the sample d escribed in detail?; 3b. Was sample size justified?; 3c. Were the groups randomised?; 3d. Was randomising appropriately done?; 3e. Was pes planus measure reliable (moderate or good); 4a. Were the outcome measures reliable?; 4b. Were the outcome measures valid?; 5a. Intervention was described in detail?; 5b. Contamination was avoided?; 5c. Cointervention was avoided?; 6a. Results were reported in terms of statistical significance?; 6b. Were the analysis method/s appropriate?; 6c. Clinical importance was reported?; 6d. Drop-outs were reported?; and 7. Conclusions were appropriate given study methods and results?. Y = yes, N = No, NA = not addressed and column coloured out if not applicable.

### Study characteristics

The study characteristics are summarised in Table 2. A range of research designs, with participants from various countries were identified for this systematic review. The studies were conducted in Korea [29, 33–35], India [28, 30], Iran [32, 36, 37], China [38] and Poland [31], published between January 2011 and July 2017.

**Table 2:** Study characteristics.

Study	N	Age (years)	Pes planus measure / diagnosis	Type of Foot Orthoses [FOs] (features)	Comparator/control	Intervention frequency
Asgaonkar and Kadam [28]	80	9.4	Foot prints (instep width and plantar arch index)	Valgus insoles (4cm thickness, rubber material)	Nil	1 year
Sinha et al. [29]	81	8.2	Symptoms (pain, fatigue and gait disturbances)	Medial Arch Orthoses (thermoplastic material, arch height dependent on patient age, foot size and alignment)	Analgesics	2 years
Pandey et al. [30]	200	> 8	Pain, Jacks test, Valgus Index, Foot Print Index and ankle ROM	Rose Schwartz insole, Thomas crooked and elongated heel with or without arch support	Foot exercise (alone/FOs)	Not addressed
Pauk and Ezerskiy [31]	130	7–15	ROM (RF, MF and FF) and ankle DF and PF	No description at all	Nil	2 years
Aboutorabi et al. [32]	50	7.87 ± 1.45	Foot Posture Index 6 (FPI-6)	Functional FOs (thermoplastic low-density PE, identical arch height to medical shoe). Medical shoe (Custom made, leather and PE with orthoses (PE shore 55).	Barefoot	Same day
Bok et al. [33]	39	10.3 ± 4.09	RCSP ≥ 4° + 1 abnormal finding on radiographs*	Customised Rigid FOs (Blake’s inverted technique)	Nil	2 years
Lee et al. [34]	20	11.0 ± 2.0	RCSP> 4° and Calcaneal pitch angle <20°.	Customised Rigid FOs (Blake’s inverted technique)	Nil	3 months
Bok et al. [35]	21	8–13	RCSP ≥ 4° + 1 abnormal finding on radiographs*	Customised Rigid FOs (inverted technique, 0°, 15° and 30°)—5mm Polypropylene and high-density EVA heel posting. Top cover = mixture of low density EVA and cork.	Shoe only	Same day
Jafarnezhadgero et al. (a)[36]	14	10.2 ± 1.4	Navicular drop > 10mm, RCSP >4° eversion and <0.31 arch height index (AHI)	Pre-fab, medially posted. Peak longitudinal height of midfoot arch is 25mm.	Nil	Same day
Jafarnezhadgero et al.(b) [37]	14	10.2 ± 1.4	Navicular drop >10mm	Pre-fab, medially posted. Peak longitudinal height of midfoot arch is 25mm.	Nil	Same day
Su et al. [38]	1	12	Not addressed	Customised insole, 7mm thickness. 9 different types (3 different arch heights 27, 30 and 33mm and 3 different material hardness 30, 35 and 40°).	Barefoot	Same day

* Bok et al. 2014 and Bok et al. 2016 –radiographic measures: Anteroposterior talocalcaneal angle (APTCA) >30 degrees; Lateral Talocalcaneal angle (LTTCA) > 45 degrees; Lateral talometatarsal angle (LTTMA) > 4 degrees; and calcaneal pitch (CP) < 20 degrees. **Abbreviations**: FOs–Foot Orthoses; ROM–Range of motion; RF–Rearfoot; MF–Midfoot; FF–Forefoot; DF–Dorsiflexion; PF–Plantarflexion; RCSP–Resting Calcaneal Stance Position; and PE–Poly Ethylene.

### Participants characteristics

The number of participants ranged from one to 200 with age ranging from seven to 15 years. Overall there were more male participants than females, with weight, height and BMI being common measurements. Ethnicity of participants was not reported by any of the studies. Participants were excluded if they had neurological, muscular or systemic diseases that may affect lower limbs and if there was a history of trauma or surgery in the past. There was no consistency in terms of the diagnostic parameters for pes planus amongst the included studies with an assortment of clinical assessments and additional evaluations, such as x-rays, used for diagnosis.

### Types of foot orthoses

While FOs were a common intervention for treating pes planus, there was great deal of variability in the parameters underpinning their use. Four studies used customised FOs [33–35, 38], two studies used pre-fabricated orthoses [36, 37], and the remaining five studies did not specify the type. Three studies used more than one type of FOs [30, 35, 38], two of those comparing the same orthoses with different levels of customisation [35, 38]. Only six studies identified the arch height of the orthoses making it easier to be replicated in clinical practice [33–38], with one study outlining the arch height to be dependent on participant’s age and foot size but no criteria were given to be followed [29]. The arch heights used across studies ranged from 25mm to 33mm. Some studies also identified the materials used and their thickness [28, 29, 32, 35, 38]. The common materials used include Polyethylene (PE) [32], 5mm thick polypropylene [35], and 4cm thick rubber [28].

### Outcome measures (OMs)

A range of outcomes and outcome measures were utilised to evaluate the effectiveness of FOs. There was a mixture of subjective (pain [28–31, 34], shoe wear pattern [30]) and objective measures (gait and radiographic parameters [28, 29, 31–33], foot print measurements [30], pressure and force distribution [32, 35, 38], arch height [31, 38], and joint moments and their asymmetry [36, 37]). The use of OMs also varied with some studies measuring the immediate effect (same day) [32, 33, 36–38] and some measuring medium term (three months) [34] and long term (1–2 years) [28, 29, 31, 33]. No study measured adverse outcomes of FOs use, other than one study identifying increased stress on joints, ligaments and cartilage with the use of 33mm arch height and 40 degree material thickness when compared to 27mm arch height and 30 degree material hardness [38]. Table 3 provides an overview of various outcomes and corresponding measures.

**Table 3:** Outcome domains and measures.

Study	Outcome domains	Outcome Measures
Asgaonkar and Kadam [28]	Pain	VAS
	Physiological Cost Index (PCI) of walking	PCI = avg.HR-basal HR/speed
	Gait parameters (step length, stride length, cadence and walking velocity)	Foot imprints along the walkway
Sinha et al. [29]	Pain (forefoot, midfoot, hindfoot)	American Orthopaedic Foot and Ankle Society (AOFAS) scores
	Foot angles*	Standardised WB radiographs
Pandey et al. [30]	Pain (midfoot, heel and calf)	Not addressed
	Gait changes	Shoe wear (less medial wear vs lateral)
	Valgus index	Foot prints
	Foot print index	
Pauk and Ezerskiy [31]	Gait pattern via GRF (Vertical, AP and ML)	Gait analysis on force platform
	Arch height (AH)	Navicular drop
	Pain	Not addressed
Aboutorabi et al. [32]	Centre of Pressure (CoP) displacements	Force plate
	Gait parameters (step length and width, walking velocity and symmetry)	
Bok et al. [33]	Resting Calcaneal Stance Position–RCSP	Clinical observation
	Radiographic measures^	Anterio-Posterior and Lateral WB radiographs of each foot
Lee et al. [34]	Pain (site, degree and frequency)	Degree with Visual Analogue Scale and frequency as weekly
	Balance (static, dynamic and functional)	A Balance motor system (computerised posturography)
Bok et al. [35]	Peak pressure (kPa)	Pedar-X-inshoe pressure system (flexible insoles, 84 capacitive sensors)
	Contact area (cm2)	
	Maximum force (N)	
Jafarnezhadgero et al. (a) [36]	Joint moment asymmetry	Gait Asymmetry index (1-(lesser moment/greater moment) x 100))
Jafarnezhadgero et al. (b) [37]	Magnitude of 3d joint moments of ankle, knee and hip.	kinetic data via gait on force plates
Su et al. [38]	Correction of foot arch	Navicular height
	Plantar pressure distribution	F-scan for plantar pressures
	Stress on foot tissue, joint cartilage and ligaments	CT scan

*Foot angles by Sinha e al. 2013: Anterio-posterior (AP) and lateral talocalcaneal (TC); AP and lateral Talo-1^st^ Metatarsal (T1MT); Lateral Calcaneal Pitch (CP); and AP Talonavicular (TN) angles.

^ Bok et al. 2014—findings on radiographs: Anteroposterior talocalcaneal angle (APTCA) >30 degrees; Lateral Talocalcaneal angle (LTTCA) > 45 degrees; Lateral talometatarsal angle (LTTMA) > 4 degrees; and calcaneal pitch (CP) < 20 degrees. Units used: kPa = kilopascal; cm^2^ = centimetre square; N = Newton and 3d = three dimensional.

### Pain

Five studies measured pain. Three studies reported statistical significant improvement in pain (p<0.05) with the use of FOs. Of these, two measured pain using Visual Analogue Scale (VAS) [28, 34] and one used American Orthopaedic Foot and Ankle Society (AOFAS) scores [29]. While the other two studies did report improvement in pain, these results were not statistically significant [30, 31].

### Foot posture measures

Clinical foot posture measures used included resting calcaneal stance position (RCSP), arch height, foot print index and valgus index. RCSP was significantly improved (i.e. reduced eversion) with FOs use compared to baseline reading (p<0.05) [33]. Two studies reported increased arch height with FOs use, one used navicular drop test [31] while the other used navicular height [38] to measure the arch height. Su et al. 2017 reported increase in foot arch height with the increase in insole arch height from 27mm to 33mm and insole material hardness from 30° to 40°. However, both these results were not statistically significant. Foot print index and valgus index measured using foot prints reduced with the use of FOs, however statistical significance of these were not reported [30].

### Gait parameters

Four studies measured changes in gait parameters including step length and width, stride length, cadence, walking velocity and symmetry and Ground Reaction Forces (GRFs) [28, 30, 31, 32]. Two studies undertook gait analysis on force platforms and both reported positive effects. There was a 0.5% decrease in the magnitude of second peak lateral force and 0.9% increase in average medio-lateral force over stance phase (p<0.05) [31] and improvement in gait symmetry and speed (p<0.05) with FOs use compared to medical shoes [32]. Another study analysed dynamic foot prints and found no statistical improvements in the gait parameters [28]. While the study by Pandey et al. 2013 [30] reported gait improvements by less medial shoe wear, but statistical significance of these results was not reported.

### Functional measures

Balance measured using computerised posturography was significantly improved in static, dynamic and functional balancing ability after three months FOs use (p<0.05) [34] and Physiological Cost Index (PCI) of walking was significantly improved using FOs (p<0.05) [28].

### Kinetic measures

Three studies measured pressure distribution. One study reported significant reduction in centre of pressure displacement using data from force plates [32]. The study by Bok et al. 2016 [35] used Pedar-X-in-shoe pressure system and reported reduction in maximum force and plantar pressures and an increase in contact area. Su et al. 2017 [38] compared effects of different arch heights and material hardness using F-scan and reported increased peak plantar pressure as the material hardness increased from 30° to 40° and at high arch height (33mm), however statistical significance of the results was not reported [38]. Bok et al. 2016 identified significantly increased contact area under medial midfoot and rearfoot, and lateral forefoot and increased maximum force under lateral forefoot, medial midfoot and lateral midfoot with the use of FOs (p<0.05) [35].

Research by Jafarnezhadgero et al. 2017 [36, 37] measured differences in joint moment asymmetry and changes in magnitude of joint moments of ankle, knee and hip joint after using FOs. The studies concluded that the use of FOs can decrease the ankle evertor moment, knee and hip abductor moments and hip flexor moment in the dominant lower limb. Furthermore, it can also result in moderate change (d = 0.54) in frontal plane subtalar asymmetry, small change (d = 0.31) in sagittal plane knee asymmetry, and moderate change in hip sagittal plane asymmetry (d = 0.65).

### Radiographic measures

Two studies measured foot angles using radiographs [29, 33], angles measured include: Talocalcaneal (TC), Talo-1st Metatarsal (T1MT), Calcaneal Pitch (CP), and Talonavicular (TN) angles. Both studies reported significant improvements in radiographic measures with use of FOs at two years.

### Summary of results

Table 4 provides a summary of results from the included studies which highlights the diversity of outcomes measured (30 different outcomes) within six broad domains. Despite this heterogeneity, the summarised findings indicate that FOs may have a positive impact across a range of outcomes. With regards to symptoms reduction, there is consistent evidence to indicate that FOs do have a positive impact in reducing pain. From an objective point of view, it appears that FOs may also have a consistent role to play in improving foot posture, gait and function. In terms of kinetic measures, many studies consistently highlight FOs positive impact across a range of measures, except for the research by Su et al (2017) and Jafarnezhadgero et al. 2017(a) which reported negative outcomes. Although these findings were not statistical significant [36, 38] and were derived from a single case study [38]. FOs also seem to positively influence structural changes in the foot anatomy over time as indicated by radiographic measurements [29, 33]. However, despite these reports of consistent positive findings, caution is required when interpreting these results due to low quality and heterogeneous evidence base. For example, only four studies in total contributed to the evidence base for functional (two studies) and radiographic (two studies) measures. Three studies of low level (case series/study) and low methodological quality contributed to the kinetic measures.

**Table 4:** Summary of the results.

Effect of FOs use	Study										
	Asgaonkar and Kadam [28]	Sinha et al. [29]	Pandey et al. [30]	Pauk and Ezerskiy [31]	Aboutorabi et al. [32]	Bok et al. [33]	Lee et al. [34]	Bok et al. [35]	Jafarnezhadgero et al.(a)[36]	Jafarnezhadgero et al.(b) [37]	Su et al. [38]
**Symptoms**											
Pain	↓(+)*	↓(+)*	↓(+)	↓(+)			↓(+)*				
**Foot posture measures**											
RCSP–eversion						↓*(+)					
Arch height				↑(+)							↑(+)?
Foot print index			↓(+)?								
Valgus index			↓(+)?								
**Functional measures**											
PCI of walking	↓(+)*										
Balance							↑(+)*				
**Gait characteristics**											
Step length	↑(+)				↑(+)						
Step width					↑(+)						
Stride length	↑(+)										
Cadence	↓(+)										
Walking velocity	↑(+)				↑(+)*						
Medial Shoe wear			↓(+)?								
Walking symmetry					↑(+)*						
**Kinetic measures**											
Plantar pressure					↓(+)*			↓(+)*			↑(-)
Maximum force (N)								↓(+)*			
Contact area (cm^2^)								↑(+)*			
Stress on tissue, joint cartilage and ligaments											↑(-)
Ankle evertor moment (Nm/Kg)										↓(+)*	
Knee abductor moment (Nm/Kg)										↓(+)*	
Hip abductor and flexor moment (Nm/Kg)										↓(+)*	
Hip abduction moment asymmetry									↓(+)*		
SP hip and knee joint moment asymmetry									↑(-)		
FP subtalar joint moment asymmetry									↑(-)		
**Radiographic measures**											
Anteroposterior talocalcaneal angle		↓(+)*				↓(+)*					
Lateral Talocalcaneal angle		↓(+)*				↔					
Lateral talo-1-metatarsal angle		↓(+)*				↓(+)*					
Anteroposterior talo-1-metatarsal angle		↓(+)*									
Calcaneal pitch		↑(+)*				↑(+)*					
Talonavicular angle		↑(+)									

↑ = increase, ↓ = decrease, ↔ = no change, (+) = positive change/improvement, (-) = negative change

* = statistical significance (p<0.05), (?) = significance not reported. **Abbreviations**: LoE: level of evidence; RCSP–Resting Calcaneal Stance Position; and PCI–Physiological cost index. **Units**; N = Newton, cm^2^ = centimetre square; NM/Kg = Newton metre per kilogram.

### NHMRC FORM framework

The analysis of results using NHMRC FORM framework is summarised in Table 5. Despite consistent positive outcomes reported in many studies, there were several factors that were unclear or inadequately addressed within the evidence base which lowered the grade of evidence. Therefore, implementation of recommendations should be undertaken with caution.

**Table 5:** NHMRC FORM framework.

Component	Grade	Comments
1. Evidence base	D–*Poor* *Level IV studies*, *or level I to III studies with high risk of bias*	Quantity: a total of 11 studies; Participants: 636 children with flexible pes planus; Level II: 3 studies; Level III-2: 2 studies; Level IV: 6 studies;
2. Consistency	C–*Satisfactory* *Some inconsistency reflecting genuine uncertainty around clinical question*	Consistent reporting of statistical significance (only two studies lacking this information); Multiple study designs; Diverse diagnostic criteria (which reflects current clinical practice standards); Heterogeneous interventions; Varied outcomes and measurements (tools and time points);
3. Clinical impact	D–*Poor* *Slight or restricted*	While nine studies reported statistical significance, only two studies reported clinical significance; Different types of FOs used with inadequate description for replicability in clinical practice; No adverse effects reported;
4. Generalisability	B–*Good* *Population(s) studied in body* *of evidence is/are similar* *to the target population*	Population studied in the evidence base is similar to the target population; Age range 7–15 years; Despite use of diverse diagnostic criteria, all studies excluded participants with other co-morbidities affecting lower limb; Studies conducted in five different countries that have different health care contexts;
Grade of recommendations	D–*Poor* *Body of evidence is weak*, *and recommendation should be applied with caution*	Overall, most studies were low level and low methodological quality; While there were some consistent and congruent findings, the current evidence base lacks clarity and uniformity in terms of diagnostic criteria, interventions delivered and outcomes measured for paediatric flexible pes planus.

## Discussion

As there continues to remain uncertainty on the effectiveness of FOs for paediatric flexible pes planus [14, 20], the aim of this systematic review was to up-date the most recent review of the literature and synthesise the current body of evidence (2011–2017). A modest body of evidence base consisting of 11 studies representing several research designs was identified. The summarised findings from this review indicate that FOs may have a positive impact across a range of outcomes including pain, foot posture, gait, function, structural and kinetic measures. Despite these consistent positive outcomes, the current evidence lacks clarity and uniformity in terms of diagnostic criteria, interventions delivered and outcomes measured for paediatric flexible pes planus. Therefore, due to the equivocal nature of the evidence base, an explicit recommendation for the effectiveness of FOs in the management of paediatric flexible pes planus cannot be made and caution is required when interpreting these findings. These findings build on the previous systematic review conducted by MacKenzie et al. (2012), which identified the growing role of FOs and its potential to positively influence a number of subjective and objective measures. While the previous review did not provide specific recommendations, this systematic review does. There are two reasons for this. Firstly, the evidence base on the effectiveness of FOs for paediatric flexible pes planus has evolved since the previous systematic review. This systematic review used a comprehensive search strategy across eight databases (compared to four in the previous systematic review) to identify research studies which formed the evidence base for this systematic review. Secondly, unlike the previous systematic review which did not use any formal means of synthesising data from the included studies, this systematic review used a widely utilised, established framework (NHMRC FORM Framework) to synthesise the evidence base and develop its recommendations. The NHMRC FORM Framework considers a range of different evidence constructs when framing a recommendation. What remains consistent with both systematic reviews, is shared concerns regarding the methodological quality of evidence base and its implication for clinical practice.

### Diagnosis of paediatric flexible pes planus

A range of diagnostic indicators for paediatric flexible pes planus were used. Most of the studies failed to identify the psychometric properties of their diagnostic methods. Common methods included RCSP [33–36], radiographic measures [33–35], range of motion (ROM) for key foot joints like ankle, FF, MF and RF [31, 32], pain [29, 30], foot print measurements [28, 32], Jack’s test [30], navicular drop [36, 37], valgus index [32] and Arch Height Index (AHI) [36]. Despite such heterogeneity, use of these diagnostic methods are supported by current literature [11, 39–41]. The variability in the diagnostic methods may be explained by the lack of an objective criteria to assess functional foot abnormalities [13].

### Types of foot orthoses

A myriad of FOs were used with minimal justification for the choice and poor description which limits replication in clinical practice. Four studies used customised FOs, of these three used inverted or Blake’s technique [33–35] with one of them comparing different inversion angles [35]. Two studies used pre-fabricated foot orthoses with medial posting and arch height of 25mm [36, 37]. Only one study provided complete information of the orthoses used including material thickness and density, description of inversion technique and top cover description [35]. Previous review on FOs efficacy for paediatric flexible pes planus also reported several different types of FOs used [14, 20]. This variability in the prescription of foot orthoses may be explained by the absence of explicit guidelines on the interventions for paediatric flexible pes planus itself [10] and the approach to prescription of orthoses specific for this population [42].

### Effect of foot orthoses on various outcomes

The use of FOs for paediatric flexible pes planus identified likely improvement across several outcome domains including subjective, objective, radiographic and kinetic data. These findings offer a different perspective to that of previously published research [14, 20] which were unable to conclude any positive impacts of FOs for paediatric flexible pes planus. There are a number of reasons which might account for this. First, since the conduct of the previous systematic reviews more than five years ago, the number of research investigating the effectiveness of FOs for paediatric flexible pes planus has increased. Second, while the previous systematic reviews focused mostly on peer-reviewed (black) literature, this systematic review searching included black and grey literature. Finally, while the previous systematic reviews used only quantitative means for data synthesis, this systematic review utilised a well-recognised framework (NHMRC FORM guide) which takes into account a range of different evidence constructs when framing a recommendation. These differences may account for the difference in conclusions.

### Limitations

As with any research, there are some limitations to this systematic review. While the systematic searching of the literature identified a modest body of evidence, there were concerns with the methodological quality. The areas of concern include sample size and sampling techniques, diagnostic criteria, development and administration of intervention and its parameters and lack of psychometrically robust outcome measures. Given that more than half of the included studies were case series/study, generalisability of the findings for these studies is limited. As flexible pes planus, can have broad ranging effects on a child, different studies focussed on different outcomes of interest. While this is to be expected, as it may reflect what occurs in clinical practice, due to the diversity of outcome measures used and heterogeneity of the interventions, a direct comparison of results between the studies was not possible. While this systematic review process was underpinned by best practice in the conduct of systematic reviews (PRISMA) [21], likely publication and language bias should be acknowledged. While strategies were implemented to avoid publication bias (such as grey literature and secondary searching), due to the complexity and imprecise nature of searching and identifying grey literature, some publications may have been missed. Due to access and resource limitations, searching was limited to English language only. Due to the comprehensive nature of the search strategy, which identified a number of studies from countries where English is not the first language, language bias was minimised but not totally avoidable.

## Conclusion

### Implications for practice

There is an increasing body of evidence to support the widely-held view that FOs may have a positive impact across a range of outcomes including pain, foot posture, gait, function and structural and kinetic measures for paediatric flexible pes planus. However, while FOs may be considered in the management of paediatric flexible pes planus, it must be recognised that the current evidence base suffers from several identified methodological concerns and therefore implementation of recommendations should be made with caution.

### Implications for future research

A modest body of evidence has identified some support for the use of FOs in the management of paediatric flexible pes planus. However significant methodological concerns of the evidence base have also been recognized highlighting the need for future research. Future research would benefit from studies that focus on developing standardised diagnostic parameters. Similarly, future research may also improve the current evidence base by developing and implementing standardised outcome measures for pediatric flexible pes planus. Finally, methodologically sound RCTs that are conducted with larger sample sizes using power calculations and include long term follow-up would assist in identifying the sustained impact of FOs on pediatric flexible pes planus. This will ensure comparison of like with like and provide unequivocal evidence for the effectiveness of FOs for pediatric flexible pes planus.

## Supporting information

S1 Appendix Prisma checklist. (PDF)

S2 Appendix Ovid AMED search. (PDF)

S3 Appendix Modified McMaster tool. (PDF)
