# Supplementary material for: The effectiveness of non-surgical intervention (Foot Orthoses) for paediatric flexible pes planus: A systematic review: Update
Source: PLoS One. 2018 Feb 16;13(2):e0193060. doi: 10.1371/journal.pone.0193060 (PMC5815602; doi:10.1371/journal.pone.0193060)
Supplement: S2 Appendix — (PDF) [file pone.0193060.s002.pdf]

S2 – Ovid AMED search

Ovid® | Wolters Kluwer

My Account | Support & Training | UniSA Library | Help | Logged in as Sindhrani Dars at University of South Australia | Logoff

Search | Journals | Books | Multimedia | My Workspace | Amirsys | EBP Tools ▾ | Mobile

▼ Search History (5) View Saved

# ▲	Searches	Results	Type	Actions	Annotations
1	("Flatf??t" or "pesplanus" or "planovalgus" or "low arch").mp. [mp=abstract, heading words, title]	454	Advanced	Display Results | More ▾	
2	("ortho*" or "insoles" or "shoe inserts" or "treat" or "non-surgical" or "therap*" or "interven*" or "manag*").mp. [mp=abstract, heading words, title]	134737	Advanced	Display Results | More ▾	
3	1 and 2	157	Advanced	Display Results | More ▾	
4	limit 3 to (child <unspecified age> and last 7 years) [Limit not valid; records were retained]	60	Advanced	Display Results | More ▾	
5	limit 4 to english language	56	Advanced	Display Results | More ▾	

Contract

Save | Remove | Combine with: AND | OR

Save All | Edit | Create RSS | View Saved

Basic Search | Find Citation | Search Tools | Search Fields | **Advanced Search** | Multi-Field Search

1 Resource selected | Hide | Change

AMED (Allied and Complementary Medicine) 1985 to August 2017
