# Supplementary material for: The effectiveness of non-surgical intervention (Foot Orthoses) for paediatric flexible pes planus: A systematic review: Update
Source: PLoS One. 2018 Feb 16;13(2):e0193060. doi: 10.1371/journal.pone.0193060 (PMC5815602; doi:10.1371/journal.pone.0193060)
Supplement: S3 Appendix — (PDF) [file pone.0193060.s003.pdf]

S3 – Modified McMaster tool

Modified McMaster Critical Review form

Fields in 'red text' have been added. Maximum score = 17 (depending on the type of study, for example, if study was not a randomised controlled trial then randomisation components were marked as NA thus changing the total score).

Assessment Components	Yes	No	Not addressed	Not Applicable
**Study Purpose**				
Was the purpose of the study clearly stated?	1			
**Literature Review**				
Was relevant background literature reviewed?	2			
**Study Design**				
RCT				
cohort				
single case design				
before and after				
case control				
cross sectional				
case study				
**Sample**	**Yes**	**No**	**Not addressed**	**Not Applicable**
Was the sample described in detail?	3			
Was sample size justified?	4			
Were the groups randomised?	5			
Was randomising appropriately done?	6			
Was pes planus measure reliable (moderate or good)	7			
**Outcomes**	**Yes**	**No**	**Not addressed**	**Not Applicable**
Were the outcome measures reliable?	8			
Were the outcome measures valid?	9			
**Intervention**				
Intervention was described in detail?	10			
Contamination was avoided?	11			
Cointervention was avoided?	12			
**Results**	**Yes**	**No**	**Not addressed**	**Not Applicable**
Results were reported in terms of statistical significance	13			
Were the analysis method/s appropriate?	14			
Clinical importance was reported?	15			
Drop-outs were reported?	16			
**Conclusions and clinical implications**	**Yes**	**No**	**Not addressed**	**Not Applicable**
Conclusions were appropriate given study methods and results?	17			
